# Measles and mumps outbreaks in Lebanon: trends and links

**DOI:** 10.1186/s12879-020-04956-1

**Published:** 2020-03-26

**Authors:** Talal El Zarif, Mohamed Faisal Kassir, Nazih Bizri, Ghida Kassir, Umayya Musharrafieh, Abdul Rahman Bizri

**Affiliations:** 1 Faculty of Medicine, Lebanese University, Beirut, Lebanon; 2 Faculty of Medicine, American University of Beirut Medical Center, Beirut, Lebanon; 3 Faculty of Medicine, University of Balamand, Koura, Lebanon; 4 Department of Family Medicine, American University of Beirut Medical Center, Beirut, Lebanon; 5 Division of Infectious Diseases, Department of Internal Medicine, American University of Beirut Medical Center, Beirut, Lebanon

**Keywords:** Measles, Mumps, Elimination, Vaccine, Lebanon

## Abstract

**Background:**

Lebanon has experienced several measles and mumps outbreaks in the past 20 years. In this article, a case-based surveillance of both measles and mumps outbreaks in Lebanon was carried out in an attempt to outline factors contributing to the failure of elimination plans and to provide potential solutions. The relationship between the outbreaks of both diseases was described and explored.

**Methods:**

A retrospective descriptive study of confirmed cases of measles and mumps in Lebanon between 2003 and 2018 collected from the Lebanese Ministry of Public Health Epidemiological Surveillance Unit public database was carried out. The information collected was graphically represented taking into consideration dates of reported cases, age groups affected, and vaccination status.

**Results:**

The mean number of measles cases was 150.25 cases/year in the 1–4 years age group, 87 cases/year in individuals aging between 5 and 14, and 63.68 cases/year in those > 14 years old. In the latter group, only 18.05% were unvaccinated. The mean number of mumps cases was 30.4 cases/year in the < 4 year age group and 53.8 cases/year in the 10–19 years age group. During the study period, every spike in measles cases was followed by a similar spike in mumps. 9.66% of measles cases occurred in individuals who received at least 2 doses of the vaccine, 52.26% in the unvaccinated, and 38% in those whose vaccination status was undetermined.

**Conclusions:**

Measles in Lebanon is a disease of the pediatric population, but adults remain at risk. Outbreaks of mumps followed those of measles and were mainly among adolescents. Presence of a large number of Syrian refugees in the country may further complicate the situation. Vaccination activities need to be intensified.

## Background

Measles is a vaccine preventable disease that is still capable of producing major outbreaks worldwide despite the availability of the vaccine [1]. Several countries have set target dates for the elimination of the disease, only to revise them later. Countries in the Eastern Mediterranean Region (EMR) had initially chosen 2010 as the target eradication date, but this has been revised twice and the current target is 2020 [2]. Measles has a high herd immunity threshold, ranging between 89 and 94%, making it more challenging for several countries to vaccinate enough individuals to reach disease elimination potential [3–5], and Lebanon is no exception in this global challenge. In 1987, the Lebanese Ministry of Public Health (LMPH) incorporated the measles monovalent vaccine in the national immunization calendar and later on, the MMR (Measles, Mumps, Rubella) vaccine was introduced in 1997 [1, 6]. The immunization schedule was later revised in March 2014 whereby children were to receive a zero dose (MCV0) at 9 months followed by a first dose of MMR (including MCV1) at 12 months and a second dose of MMR (including MCV2) at 18 months.

In 2018, UNICEF sent an appeal to its donors to help close the gaps in vaccine funding, a key factor that is further crippling the situation in Lebanon [7]. Achieving a high level of population immunity to protect against the spread of measles can only be reached via administering two doses of the current measles vaccine. Measles antigen-containing vaccine 1 (MCV1), the first measles vaccine, is usually administered as part of routine vaccination services, while MCV2 can be received through the same services or through mass vaccination campaigns [8]. The estimated global coverage with the MCV1 has seen a significant rise from 72% in 2000 to 85% in 2010 [9], yet Lebanon is still amongst the countries with < 90% coverage with MCV1 [10]. The ease of transmission of the disease, its free circulation through travelers, and the mass displacement of refugees from conflict areas, mainly from Syria, to Lebanon coupled with low MCV1 coverage have made the goal of elimination more challenging [8, 11, 12].

According to the WHO-UNICEF estimates, the percentage of MCV1 and MCV2 vaccinated individuals during 2018 were at 82 and 63% respectively implying that Lebanon is still far from reaching the elimination threshold set at 95% [10]. Compulsory vaccination for children prior to enrollment in public schools in Lebanon was mandated by the government in 2013; however, in a recent survey conducted at the national level, children aged 12–59 months were found to have MCV1 vaccine coverage of 86.7% and MCV2 coverage of 64.2%, [13].

The fact that the measles vaccine is widely available in the form of a combination vaccine with mumps and rubella implies that while attempting at eliminating measles, countries would be targeting mumps and rubella as well; in fact, it has been suggested that a booster dose of MMR will aid in prevention of mumps outbreaks among adolescents [14]. Besides, a mathematical model for measles and rubella transmission has shown that in the endeavor to meet the goal of measles elimination, countries would inadvertently eliminate rubella which has significantly lower transmissibility [3].

Data from the LMPH Epidemiological Surveillance Unit (ESU) reveal that measles outbreaks are still occurring in Lebanon. Isolated strains included D4 (during 2003–2007), D8, B3, and H1 (during 2013) [6]. This requires a careful approach that starts by delineating the recent trend of measles occurrence in Lebanon in an attempt to understand the level at which the elimination plan is not working [15]. Besides measles, we also address the incidence of mumps in Lebanon, given that they both belong to the Paramyxoviridae family and that both share the MMR combination vaccine. This article also provides several recommendations aimed at pushing the Lebanese efforts closer to achieving standards compatible with measles and mumps elimination.

## Methods

This study is a retrospective descriptive analysis of all cases of measles and mumps reported to the LMPH-ESU database between 2003 and 2018. Data relevant to the number of cases, age and vaccination status were collected in regard to both viruses.

### LMPH-ESU public database and laboratory network

Established at the end of 2001, the ESU receives input from medical centers, dispensaries, schools, public and private hospitals and MOPH/ESU caza teams conducting active surveillance. The Central Public Health laboratory (2002–2007) and Rafic Hariri University Hospital laboratory (2008-present) are the national measles laboratories. The Central Public Health Laboratories in Sultanate Oman and the Pasteur Institute in Tunisia are the regional reference laboratories to support measles and rubella elimination and optimize surveillance programs in the EMR.

### Measles and mumps data collection

Data on documented measles and mumps cases in Lebanon were collected from the publicly available LMPH-ESU database, which anonymously keeps track and reports these cases based on laboratory, epidemiologic, and clinical grounds. Physicians and health institutions are obliged to report cases to LMPH by filling an individual-reporting form or a specific measles/rubella case-based form.

### Working definitions

The measles cases reported by the LMPH are classified as being either of two categories: (1) laboratory-confirmed cases based on positive measles IgM titers and/or positive RT-PCR results or (2) epidemiologically-confirmed cases that lacked laboratory confirmation at the time of documentation but had been in direct contact with lab-confirmed cases within the last 28 days and fulfilled the clinical criteria of measles diagnosis. Any case that does not belong to either of these two categories was discarded after also confirmaing via lab testing (IgM titers or RT-PCR) that it was not a rubella case which presents with a similar clinical picture of fever and maculopapular rash but is milder and associated with lymphadenopathy. As for mumps, the cases were also confirmed using laboratory testing which included isolation of the mumps virus from a clinical specimen, detection of seroconversion or a significant rise (at least fourfold) in serum mumps IgG titer, or positive serological testing for mumps–specific IgM antibodies in the absence of mumps immunization in the preceding 6 weeks.

### Data analysis

**Fig. 1:**
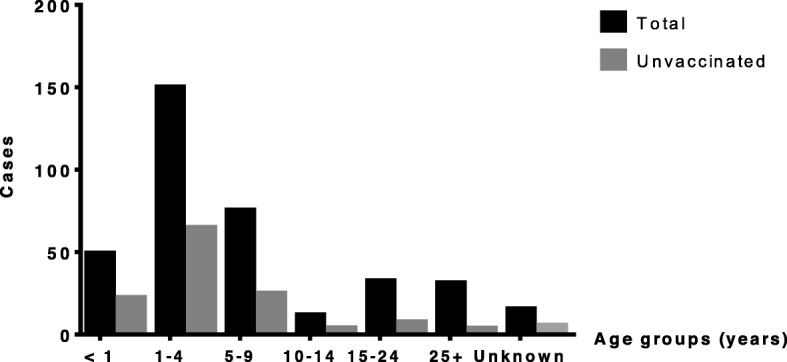
Distrubtion of mean number of measles cases between 2003 and 2018

**Fig. 2:**
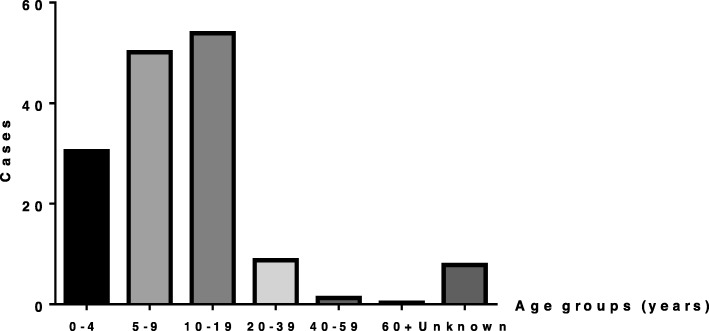
Distribution of mumps cases among different age groups between 2003 and 2018

**Fig. 3:**
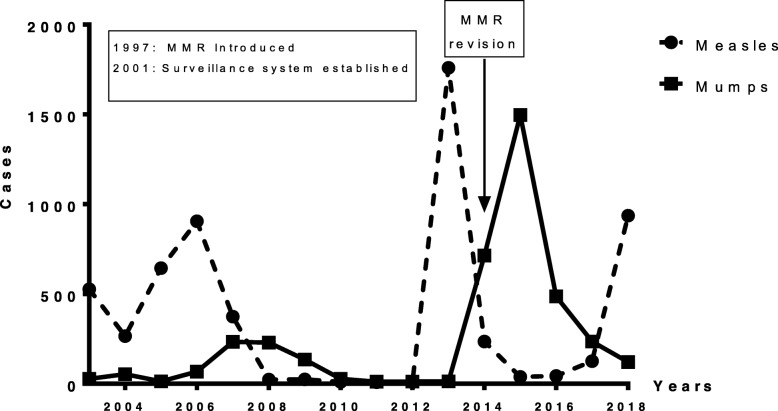
Timeline of total number of cases of mumps and measles between 2003 and 2018 with milestone interventions denoted

**Fig. 4:**
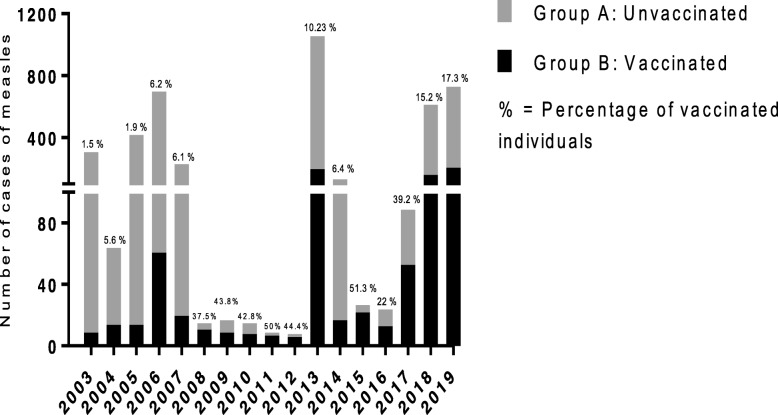
Vaccination status of individuals who contracted measles in the period between 2003 and 2018

Collected data on the incident measles and mumps cases were plotted on a Microsoft Excel spread sheet and analyzed using GraphPad Prism 6. The reported measles and mumps cases between 2003 and 2018 were categorized by age to identify the age group most susceptible to develop the disease (Figs. 1 & 2). Measles cases were then plotted in the same graph as the mumps cases (Fig. 3) in order to demonstrate the temporal relationship between the outbreaks of each disease amongst the Lebanese population. Measles cases were also plotted according to vaccination status (Fig. 4). Cases with unspecified vaccination status were discarded from the analysis.

## Results

### Age distribution of measles and mumps

The mean number of measles cases occurring in Lebanon between 2003 and 2018 was calculated and distributed over 7 age groups as shown in Fig. 1. The mean number of cases in the < 1-year age group is 49.1 cases/year (13.45%), of which 22.43 cases/year are unvaccinated. This number increases to a maximum of 150.125 cases/year (41%) in the 1–4 years age group, of which 64.93 cases/year are unvaccinated. Beyond this group, there is a decline in the number of cases with increasing age reaching 11.875 cases/year in the 10–14 years age group; however, this number gradually increases again. The mean number of measles cases in individuals older than 14 years is equal to 63.68 cases/year (17.41%) where only 18.05% are unvaccinated cases. A further detailed representation of the trend of annual number of cases of measles in Lebanon is expressed in Table 1.

**Table 1:** Annual number of total and unvaccinated measles cases in Lebanon between 2003 and 2018 stratified by age groups

Age groups (years)	< 1 yrs	< 1 yrs	1–4 yrs	1–4 yrs	5–9 yrs	5–9 yrs	10–14 yrs	10–14 yrs	15–24 yrs	15–24 yrs	25+ yrs	25+ yrs	N/A	N/A
2003	54	44	174	130	48	25	11	4	117	29	15	4	53	30
2004	10	5	68	20	26	6	5	2	84	14	6	2	15	1
2005	78	59	287	194	139	75	18	7	37	14	14	8	45	19
2006	97	75	364	219	201	95	42	21	129	54	49	27	71	20
2007	35	30	156	86	120	44	18	6	11	2	12	2	21	13
2008	4	2	10	0	4	0	1	0	2	1	2	1	1	0
2009	5	5	4	1	6	1	0	0	1	1	0	0	0	0
2010	5	3	5	2	0	0	0	0	2	1	2	1	0	0
2011	3	2	5	0	1	0	0	0	0	0	1	0	0	0
2012	4	1	3	2	0	0	2	1	0	0	0	0	0	0
2013	251	9	691	93	378	60	49	5	85	4	281	7	25	2
2014	41	4	91	7	55	4	7	0	14	0	22	0	5	0
2015	6	1	21	11	6	2	3	3	1	1	2	2	0	0
2016	10	1	19	5	12	5	1	0	3	0	4	0	1	0
2017	24	15	38	14	23	4	5	2	13	0	23	1	4	0
2018	160	103	466	255	186	79	28	13	20	1	67	7	9	4
Average	49.2	22.4	150.1	64.9	75.3	25.0	11.9	4.0	32.4	7.6	31.3	3.9	15.6	5.6

: Total cases: Unvaccinated cases

The mean number of mumps cases occurring in Lebanon between 2003 and 2018 was similarly calculated and distributed over 7 age groups as shown in Fig. 2. The mean number of cases in the 0–4 year age group is equal to 30.4 cases/year. This number increases to a maximum of 53.8 cases/year in the 10–19 years age group. Beyond this group, there is a steady decline in the number of cases with increasing age; the number of cases reaches 0.31 cases/year in the 60+ years age group. A further detailed representation of the trend of annual number of mumps cases in Lebanon is expressed in Table 2.

**Table 2:** Annual number of mumps cases in Lebanon between 2003 and 2018 stratified by age groups

Age groups (years)	0–4	5–9	10–19	20–39	40–59	60+	N/A
2003	12	18	5	3	0	0	1
2004	17	102	41	8	1	0	15
2005	6	9	7	3	0	0	3
2006	8	21	19	5	0	0	1
2007	5	2	4	3	0	0	0
2008	18	24	14	7	2	0	3
2009	35	94	81	10	1	2	10
2010	28	71	113	12	1	1	3
2011	41	60	26	5	0	0	3
2012	10	8	8	2	0	0	0
2013	6	4	1	1	0	0	0
2014	5	5	2	1	0	0	0
2015	8	3	3	0	0	0	0
2016	91	267	309	36	5	0	6
2017	51	4	1	0	0	0	1
2018	77	140	184	37	6	1	41

### Measles and mumps relationship

In Fig. 3, the number of cases of measles and mumps occurring in Lebanon were compared head to head between the years of 2003 and 2018. Five hundred twenty-seven cases of measles were reported in 2003. This was followed by an increase in the number of mumps cases from 28 in 2003 to 54 in 2004. The number of measles cases dropped by half in 2004 and later resurged in 2005 and 2006 to 644 and 905 cases respectively. This was once again concomitant with a rise in mumps cases in 2007 and 2008 to 233 and 229 respectively. Beyond that, the number of both mumps and measles cases was almost negligible until an alerting peak in measles cases took place in 2013 recording a staggering 1760 cases which was once again followed by a surge in mumps cases in 2015 to 1496 cases. Finally, both measles and mumps cases decreased dramatically in the following years until another rise in measles cases which reached 938 cases in 2018 with only 121 mumps cases in the same year. The most recent rise in measles cases has emerged despite the revision in the MMR national immunization schedule which was instituted in 2014, as shown in Fig. 3.

### Measles cases distribution according to vaccination status

Figure 4 shows the distribution of individuals who contracted measles into two groups based on their vaccination status: Group A comprises the unvaccinated individuals while Group B includes all the vaccinated individuals. The percentages of these groups were then calculated and plotted on the graph. The results show that among those who contracted measles during the period between 2003 and 2018, the percentage of individuals vaccinated against the virus averaged at 9.66% while a mean of 52.26% of the cases were confirmed to be non-vaccinated. 38% of the cases had an unknown vaccination status.

## Discussion

### Epidemiology of measles and mumps in Lebanon

The literature is scant regarding the epidemiology of measles and mumps in Lebanon. The above data clearly portray that Lebanon has experienced several measles outbreaks in the past two decades. Given the < 90% MCV1 coverage rate and the lower MCV2 coverage, Lebanon is still far from achieving the goal of measles elimination and is at risk of experiencing new outbreaks, especially with the decline in the proportion of seropositive young adults over the past 15 years. In 2011, Chamat et al. assessed the measles and mumps antibody titers of 502 medical and paramedical students in Lebanon showing a decline in seropositivity which equaled 86% for measles and 76% for mumps; this was attributed to the decreased incidence of both diseases [16]. In 2016, Ozaras et al. described the impact of the neighboring Syrian crisis on the Lebanese public health scene; the influx of more than 1 million Syrian refugees living in poor sanitory conditions into Lebanon had created a favorable medium for the circulation of multiple infectious diseases [11]. This might explain increases in the number of measles and mumps cases in 2013 and 2014 respectively, as seen in Fig. 3. Fortunately, the LMPH reacted and initiated a national immunization campaign in 2014 [11]. In 2019, Kmeid et al. evaluated the vaccination status of 571 Syrian and Lebanese children, showing low compliance with the measles vaccine (55–70%) and a higher compliance with the MMR vaccine (96–100%). Socioeconomic factors seemed to play a major role in vaccination compliance [17], while contracting measles has been itself associated with a significant economic burden [18].

As for morbidity, previous measles outbreaks in Lebanon were associated with a case fatality rate of 2 per 1000 reported cases [6]. It has been revealed that new strains like the B3 strain, which is a more transmissible genotype, are becoming increasingly widespread leading to new epidemics worldwide [19]. Data in Lebanon showed that the measles strains in 2013 were B3, D8, and H1 while mumps strains are yet to be identified [6].

### Age distribution of measles & mumps and current vaccine protocol

In Fig. 1, it is clear that children aged between 1 and 4 years are the most susceptible individuals to contracting measles. Early in life, children usually acquire passively transferred maternal antibodies providing them with immunity against the virus. These antibodies are usually cleared within 6 months from the newborn’s serum, and as a result, children become more susceptible to infection. There is no data in Lebanon that would help assess the seropositivity of women of childbearing age against measles or mumps. However, children from immunized mothers may still be susceptible to infection despite maternal immunization since maternal antibody levels can be affected by certain factors like prematurity and declining maternal immunity due to decreased exposure to wild-type viruses [20, 21].

The Center for Disease Control (CDC) recommends routine vaccination of children with the measles-mumps-rubella (MMR) vaccine in a 2-dose series scheduled at 12–15 months and 4–6 years with the possibility of giving the 2nd dose as early as 4 weeks after the 1st one [22]. The WHO recommends that the first dose be given at 9 months when attack rates are high and risk of serious disease among infants exists. In low risk areas, the first dose can be administered at 12–15 months. Although the second dose is generally administered at school age (4–6 years), it may be given as early as 1 month following the 1st dose, depending on the measles status in the country [23]. In Lebanon, the LMPH adopted a vaccination strategy where children will receive a zero dose (MCV0) at 9 months followed by a first dose of MMR (MCV1) at 12 months and a second dose of MMR (MCV2) at 18 months [24]. This appears to be well warranted given the significant number of measles cases in the younger ages as seen in Fig. 1.

It is clear that measles is a disease of the pediatric population in low vaccination areas, but adult cases must not be overlooked (Fig. 1). A recent study by the WHO revealed that almost 77% of the potentially preventable cases of measles were among children aging less than 15 years. Although the average number of measles cases in individuals older than 14 years in Lebanon (Fig. 1) is much less than the 45% reported by the European CDC database in 2017 for the same age group, it remains a significant percentage that should not be ignored [25]. A study implemented in China revealed that the seropositivity rate of measles antibodies was significantly lower in subjects aged 15–19 years than those aged 5–9 years. This result was attributed to the waning antibody titers especially that there are no circulating wild-type viruses to confer natural immunity. It is rather challenging to implement a vaccination campaign to target adults. It would be more feasible to conduct an immunization program that involves high school students when they are still in mandatory education. This will protect those teenagers from future measles infections and will contribute to the protection of their children via increasing the measles maternal antibody levels.

As can be seen in Fig. 2, it is evident that mumps cases are most commonly observed in children and adolescents. In a recent study by Cardemil et al., there was a call for a third shot of MMR to control mumps outbreaks in university students in the face of waning immunity [14]. It is reasonable to assume that the resurgences of the mumps outbreaks in Lebanon for those who are 10–19 years old could be attributable to waning immunity [14]. Given this common theme of waning immunity, revaccination with MMR should be encouraged in an attempt to adopt a strategy that will aid in measles and mumps elimination and prevention of the Congenital Rubella Syndrome. In one study, revaccination of secondary school students regardless of previous measles vaccination or disease status resulted in complete protection, raising seropositivity from 91 to 100% making it a very promising initiative [26].

### Measles and mumps relationship

It appears from Fig. 3 that there is a close relationship between the number of incident measles and mumps cases in Lebanon, and a clear pattern can be noticed when examining the outbreaks of each of these diseases. Over the past 20 years, after every increase in measles cases among the Lebanese population, a parallel increase – although less intense – in mumps cases can be observed 1 or 2 years later. This trend has not been established elsewhere in the literature, yet it might be expected as the two diseases share a common vaccine. Should the rise in measles cases reflect a shortfall in the vaccination strategy, it would be logical to expect a deficiency in the immunity against mumps as well, and thus mumps outbreaks paralleling the measles outbreaks. Taking into consideration that the mumps vaccine is less immunogenic than that of measles [27], this might be of immense importance for public health strategies, as it might help anticipate any mumps epidemic before it occurs. For example, Israel witnessed an outbreak of 262 mumps cases between January and August 2017 although vaccination levels reached ≥96% [28]. This was concomitant with a measles outbreak during the summer of the same year [29]..

It is important to note that measles outbreaks will have long-lasting impacts that could be explained by measles-induced immune damage. Epidemiological studies from the USA, Denmark and England back in 1940, found that rates of non-measles infectious disease mortality were tightly coupled to measles incidence—with a greater mortality rate when the incidence of measles was higher. Using computer models, measles infection was found to predispose children to all other infectious diseases for up to a few years, due to reduction in host resistance following measles infection that may extend over a period of more than 2 years [30]. In vitro and in vivo studies attributed this to the immunosuppression effects of measles that caused depletion of B and T lymphocytes. The effect is more on the memory than naïve cells in the case of T cells [31, 32] but it is equal on both memory and naïve B cells [31, 33]. Thus, after fighting off measles, the immune system makes a comeback but has ‘forgotten’ what it had once learned. The child’s immune system has to start afresh, rebuilding immune protection against viruses and bacteria it had previously fought off.

While we are still far from defeating measles, we can use the measles epidemics to review our vaccination strategies, reassess the level of herd immunity, and prepare our healthcare systems for an imminent mumps epidemic as per the established pattern. In light of the rising number of measles cases in 2018, the LMPH has established an outbreak control strategy via increasing training sessions for health centers, hospitals, and ESU teams, spreading public awareness through publishing a weekly bulletin on measles surveillance, sending out posters and other advocacy materials, and initiating a massive media campaign. Immunization activities with the MMR vaccine in vulnerable areas were conducted to help curb the ongoing measles outbreak and help prevent possibly incoming mumps outbreaks [34].

### Measles cases distribution according to vaccination status

Figure 4 shows that most of the measles cases were unvaccinated. Multiple factors might affect the accuracy of these figures and numbers. One factor that must be taken into consideration is the number of vaccine doses administered; many of those who claimed they contracted measles despite vaccination might have received an insufficient dose of the vaccine. This misunderstanding might thus skew the percentages and falsely elevate the numbers of those contracting measles despite vaccination. Only 61.92% of the cases had a documented vaccination status, which still lags behind the target set by the WHO at a minimum of 80%, indicating a far-from-sufficient immunization system [15]. The possibility that non-vaccinated persons are clustered together should be considered, implying that the estimated vaccination coverage rates do not reflect the status of the general population but rather represent that of a higher risk subpopulation [35].

Referring to Fig. 4, 52.26% of people who contracted measles were unvaccinated. A recent study by Mansour et al., found several factors that hinder vaccination, and these include socio-demographics, as well as knowledge, beliefs and practices associated with age-appropriate vaccination [36]. Mothers may perceive that the vulnerability to disease lessens with older age considering the first dose as the main source of protection whereas boosters are regarded as add-ons. Subsequent non-compliance will result and their children will be lost to follow up [37].

The current used vaccine in Lebanon is an-attenuated live measles vaccine that belongs to the Schwartz strain according to the WHO [6]. As mentioned earlier, individuals in Lebanon get a total of 3 doses of vaccine against measles. National catch-up campaigns against measles are conducted in case of any outbreaks as was the case in 2001, 2008 and 2013 in an attempt to enhance vaccination coverage and reduce the number of susceptible individuals. In an American study that discussed one of the largest measles outbreaks among highly vaccinated students, whose source case had coincidentally contracted the virus from Lebanon, it was revealed that students who had received both doses of the vaccine outside the United States were more susceptible to the infection than those who received both doses in the United States [38]. This was partly attributed to the quality of storage of the vaccine (the cold chain). Measles vaccine should be stored at 2–8 °C as improperly stored vaccines may fail to provide protection against the disease. The quality of vaccine storage should be reviewed in Lebanon in lights of our results. Besides, immunological and genetic research is recommended to evaluate vaccine effectiveness against the aforementioned strains and identify any new ones that might not be covered by the available vaccines [19].

### Recommendations for measles prevention

The presented data should serve as a guide for the strategies that must be followed in the current fight against measles. The WHO has published a global strategy which can serve as the base upon which we build our own national strategy [2]. Active surveillance of measles contacts should be implemented in case national vaccination campaigns are not possible, regardless of the vaccination status; in fact, secondary measles contraction in vaccinated individuals can present itself with symptoms dissimilar to those typical of the disease, allowing viral circulation in the absence of active monitoring [29]. This has been the case during the Israeli outbreak where the primary case presented with only fever and rash which made it quite challenging to suspect measles [29]. More to the point, the surveillance system must also improve in tracking vaccination through encouraging families to hold onto home-based records [15]. This is to be bolstered by routine re-vaccination of high-risk individuals including health-care workers and contacts of measles cases in a strategy similar to the ring vaccination adopted in eradicating other viruses like the Pox virus; this would help limit the dissemination of the virus and lead to eventual elimination [19].

Several infectious disease outbreaks were noted in Lebanon in correlation with the huge influx of Syrian refugees into the country [11, 39]. Data from Syria have shown subpar levels of first dose coverage of the measles vaccine ranging between 50 and 79% among their population [8]. The Italian experience showed that the migrant communities are not necessarily representative of their source population when it comes to measles vaccine coverage rates [40]. The discrepancy between migrants and their source population reflects the need for special screening and vaccination campaigns in migrants and refugees in Lebanon.

One of the cornerstones in the strategy to eliminate measles will be building public trust in the measles vaccine. Ever since the spread of the later-falsified MMR-Autism theory, measles vaccination rates witnessed a hefty drop in some regions in the Western world. The UK was declared endemic for measles in 2008, with some areas of London and Ireland reaching a vaccination level of only 60%. The United States also witnessed several outbreaks in the current decade with vaccination levels as low as 50% - far from the 95% herd immunity threshold [41]. Although the advocates of this theory are decreasing, some parents are still exhibiting a general anti-immunization approach [42]. Effective communication teams should be created and invested in to target different audiences and inform them on the importance of vaccination and the dangers of unvaccination [2]. This is highlighted in a recent study by Hoffman et al., which warned that social media outlets may facilitate anti-vaccination by enabling the diffusion of century old arguments and techniques and facilitating anti-vaccination behaviour [43].

Last but not least, investing in local capacity building and research projects to understand the epidemiology and behaviour of the measles virus in relation to the population dynamics in Lebanon is of immense importance to establish national strategy and guidelines [6].

### Recommendations for mumps prevention

It is essential that clinicians and individuals are made aware of any ongoing mumps outbreaks to increase suspicion and reporting of the disease. The LMPH must arrange awareness campaigns in national high schools and universities to educate adolescents on the clinical presentations and complications of mumps. High school and college students should be encouraged to maintain adequate hygiene by washing hands before meals and after toilet use and minimizing contact with their peers [44]. In the face of outbreaks, the effectiveness and feasibility of booster vaccination campaigns must be considered. Cardemil et al. suggest that a booster MMR dose helps prevent mumps cases among adolescents [14]. This was later backed up with evidence of the possible contribution of a third dose of MMR in preventing mumps infection in military recruits with waning antibody titers [45], especially that a study by Lewnard et al. showed that protection from the mumps vaccine lasts for 27 years on average [46]. Principi et al. acknowledge the effectiveness of booster doses in controlling outbreaks but argue that it is not clear whether these boosters can actually prevent them as the protection they provide wanes over time [47]. Another hurdle to be considered is the emergence of new mumps strains that are resistant to already established vaccines; it is thus recommended to maintain an active surveillance of circulating strains so that new vaccines including newer mumps strains are developed [47].

### Limitations

Our study is a retrospective descriptive analysis based on data gathered by LMPH-ESU about reported cases of measles and mumps. It lacks key information regarding the seroprevalence of measles and mumps in the whole population and in specific groups: mainly women of child-bearing age. Unfortunately, data showing measles cases with only one dose of the measles vaccine is also lacking, and the presence of a high percenetage of measles cases with unknown vaccination status remains a major issue that may have affected and skewed our analysis. As for mumps, no information was available about the circulating strains and vaccination status of those affected.

## Conclusions

Measles in Lebanon is a disease of the pediatric population, but adults remain at risk. Outbreaks in measles were characteristically followed by mumps outbreaks which were mainly among adolescents. Several factors such as the Syrian conflict and large influx of refugees to the country may be contributing factors to the emergence of these outbreaks. Vaccination activities against measles and mumps need to be intensified to achieve higher immunity levels. The use of a third dose of mumps vaccine should be further explored in the setting of waning antibody titers. Vaccine quality and storage have to be monitored to ensure efficacy. A clear national plan must be set and followed in order to finally achieve the goal of measles and mumps elimination.

## Data Availability

The datasets generated and analysed in this study are available in the Lebanese Ministry of Public Health Epidemiological Surveillance Unit public records. Public access to the databases is open and requires no permission. These can be accessed at: https://www.moph.gov.lb/en/Pages/2/193/esu.

## Abbreviations

LMPH: Lebanese Ministry of Public Health
ESU: Epidemiological surveillance unit
MCV: Measles antigen-containing vaccines
MMR: Measles-mumps-rubella
CDC: Center for disease control
EMR: Eastern Mediterranean Region
